# Enhancing predictability of *IDH* mutation status in glioma patients at initial diagnosis: a comparative analysis of radiomics from MRI, [^18^F]FET PET, and TSPO PET

**DOI:** 10.1007/s00259-024-06654-5

**Published:** 2024-02-24

**Authors:** Lena Kaiser, S. Quach, A. J. Zounek, B. Wiestler, A. Zatcepin, A. Holzgreve, A. Bollenbacher, L. M. Bartos, V. C. Ruf, G. Böning, N. Thon, J. Herms, M. J. Riemenschneider, S. Stöcklein, M. Brendel, R. Rupprecht, J. C. Tonn, P. Bartenstein, L. von Baumgarten, S. Ziegler, N. L. Albert

**Affiliations:** 1 Department of Nuclear Medicine, University Hospital, LMU Munich, Marchioninistr. 15, 81377 Munich, Germany; 2 Department of Neurosurgery, University Hospital, LMU Munich, 81377 Munich, Germany; 3 Department of Neuroradiology, Klinikum Rechts der Isar, Technical University of Munich, Munich, Germany; 4 German Center for Neurodegenerative Diseases (DZNE), 81377 Munich, Germany; 5 Center for Neuropathology and Prion Research, Faculty of Medicine, LMU Munich, Munich, Germany; 6 Department of Neuropathology, University Hospital Regensburg, 93053 Regensburg, Germany; 7 Department of Radiology, University Hospital, LMU Munich, 81377 Munich, Germany; 8 Munich Cluster for Systems Neurology (SyNergy), 81377 Munich, Germany; 9 Department of Psychiatry and Psychotherapy, University of Regensburg, 93053 Regensburg, Germany; 10 German Cancer Consortium (DKTK), Partner Site Munich, German Cancer Research Center (DKFZ), 69120 Heidelberg, Germany; 11 Bavarian Cancer Research Center (BZKF), 91054 Erlangen, Germany

**Keywords:** Radiomics, FET PET, TSPO PET, BraTS, Glioma, *IDH* mutation status

## Abstract

**Purpose:**

According to the World Health Organization classification for tumors of the central nervous system, mutation status of the isocitrate dehydrogenase (*IDH*) genes has become a major diagnostic discriminator for gliomas. Therefore, imaging-based prediction of *IDH* mutation status is of high interest for individual patient management. We compared and evaluated the diagnostic value of radiomics derived from dual positron emission tomography (PET) and magnetic resonance imaging (MRI) data to predict the *IDH* mutation status non-invasively.

**Methods:**

Eighty-seven glioma patients at initial diagnosis who underwent PET targeting the translocator protein (TSPO) using [^18^F]GE-180, dynamic amino acid PET using [^18^F]FET, and T1-/T2-weighted MRI scans were examined. In addition to calculating tumor-to-background ratio (TBR) images for all modalities, parametric images quantifying dynamic [^18^F]FET PET information were generated. Radiomic features were extracted from TBR and parametric images. The area under the receiver operating characteristic curve (AUC) was employed to assess the performance of logistic regression (LR) classifiers. To report robust estimates, nested cross-validation with five folds and 50 repeats was applied.

**Results:**

TBR_GE-180_ features extracted from TSPO-positive volumes had the highest predictive power among TBR images (AUC 0.88, with age as co-factor 0.94). Dynamic [^18^F]FET PET reached a similarly high performance (0.94, with age 0.96). The highest LR coefficients in multimodal analyses included TBR_GE-180_ features, parameters from kinetic and early static [^18^F]FET PET images, age, and the features from TBR_T2_ images such as the kurtosis (0.97).

**Conclusion:**

The findings suggest that incorporating TBR_GE-180_ features along with kinetic information from dynamic [^18^F]FET PET, kurtosis from TBR_T2_, and age can yield very high predictability of *IDH* mutation status, thus potentially improving early patient management.

**Supplementary Information:**

The online version contains supplementary material available at 10.1007/s00259-024-06654-5.

## Introduction

Gliomas are the most common primary brain tumors, accounting for a significant proportion of central nervous system malignancies in adults [1]. They are characterized by their heterogeneity in terms of histology, genetics, and clinical behavior, which presents a serious challenge for accurate diagnosis, prognosis, and treatment planning [2]. In recent years, molecular markers have emerged as critical prognostic and predictive factors in glioma patients [3]. One of the most important molecular alterations is a spectrum of mutations in the isocitrate dehydrogenase (*IDH*) genes, particularly *IDH1* and *IDH2* [4–6]. *IDH*-mutant gliomas are associated with a more favorable prognosis and better response to certain targeted therapies compared to their *IDH* wild-type counterparts [7–10]. Therefore, *IDH* mutation status has been incorporated into the latest World Health Organization (WHO) classification of central nervous system tumors (WHO CNS 5) as a key molecular marker [3]. *IDH* mutation status is typically determined through molecular testing on tissue samples obtained invasively by stereotactic biopsy or surgical resection. However, since these procedures carry inherent risks, non-invasive methods for predicting *IDH* mutation status might present a valuable tool in early patient management [11–14]. Thus, non-invasive prediction of *IDH* mutation status holds significant potential for tailoring personalized patient care strategies.

Among the non-invasive imaging modalities, positron emission tomography (PET) and magnetic resonance imaging (MRI) have shown promise in providing valuable information about glioma biology. MRI, with its superior soft tissue contrast, is the diagnostic gold standard providing information on lesion composition and extent [15]. Contrast-enhanced (CE) T1-weighted MRI is routinely used for tumor visualization, since, for example, the presence of ring enhancement is indicative of a fast-growing glioblastoma exhibiting blood-brain barrier disruption around necrotic tissue in the tumor center [16]. Additionally, T2-weighted MRI sequences reveal edema and tumor invasion into the surrounding brain parenchyma, further aiding in glioma characterization [17]. However, MRI lacks sensitivity and specificity for describing biological properties on a molecular level. This can be improved using PET imaging with radiolabeled tracers allowing to visualize and quantify specific molecular targets in vivo, such as amino acid metabolism, cellular proliferation, and inflammation [18]. Amino acid PET using [^18^F]FET, for instance, shows elevated uptake in regions of increased amino acid metabolism, such as active tumor tissue and infiltrative tumor margins [19]. Aside from delineating active tumor, [^18^F]FET PET has proven its utility in identifying intratumoral heterogeneity and differentiating tumor recurrence from pseudoprogression [20, 21]. Despite being less tumor-specific, PET imaging using radioligands targeting the translocator protein (TSPO), which is upregulated not only in tumor cells but also in activated microglia or macrophages, has demonstrated correlations with histologic tumor grade, specific transcriptional glioma subtypes, and survival [22–27]. A recent study revealed that there is no correlation between PET information and relative contrast enhancement on T1-weighted MRI [28]. This finding suggests that the blood-brain barrier permeability, as assessed using CE MRI, appears not to be the driving factor for specific PET signal of [^18^F]FET and [^18^F]GE-180 PET. Moreover, the study demonstrated the complementary nature of these imaging modalities.

The diagnostic relevance of the different modalities for glioma assessment is increasingly being exploited by the application of radiomic analyses [29]. Radiomic features may provide valuable information about tumor phenotypes and microenvironment by reflecting various aspects of tumor biology, such as shape, heterogeneity, vascularity, or cellular density. PET- and MRI-derived radiomics have been successfully employed in various applications [29].

Given the potential of PET, MRI, and radiomics in glioma assessment, this study aimed to compare and evaluate the predictive value of radiomics derived from TSPO PET, static and dynamic [^18^F]FET PET, and T1- and T2-weighted MRI for determining the *IDH* mutation status in glioma patients at the time of initial diagnosis. We hypothesized that specific radiomic features derived from different imaging modalities could serve as biomarkers for *IDH* mutation status prediction, allowing for non-invasive and accurate molecular classification of gliomas. Our findings contribute to the growing body of evidence on the potential of multimodal imaging and radiomics in improving the management of glioma patients, with the ultimate goal of facilitating personalized treatment strategies.

## Material and methods

### Patients

In this study, glioma patients at initial diagnosis who received dual PET and MRI scans prior to any therapeutic intervention were included consecutively. Multimodal imaging included TSPO PET using [^18^F]GE-180, amino acid PET using [^18^F]FET, T1-weighted MRI with and without contrast agent, and T2-weighted MRI. All scans were performed before any therapeutic intervention with on average 4 ± 6 days between both PET scans and 9 ± 10 days between PET and MRI scans. Tissue samples for histopathological and molecular genetic classification (e.g., mutation of the *IDH1/2* gene, codeletion of chromosomes 1p and 19q) were obtained using either stereotactic biopsies or tumor resection. Biopsy extraction was conducted with the guidance of imaging techniques, also taking into account PET information. The classification was performed according to the WHO grading system for tumors of the central nervous system revised in 2021 [3]. The tissue samples extracted at the Department of Neurosurgery of the LMU University Hospital, LMU Munich, were evaluated at the Center for Neuropathology and Prion Research, LMU Munich, and the Department of Neuropathology of the University Hospital Regensburg.

Furthermore, TSPO binding affinity status was derived by assessing the presence of a polymorphism in the gene encoding the TSPO. The genotyping was performed at the Department of Psychiatry of the University Hospital Regensburg as previously described [30], allowing for a categorization of the patients as low-, mixed-, or high-affinity binders (LAB, MAB, HAB) [31–33].

All patients gave written informed consent to the data analysis. The study was approved by the local ethics committee (Ethikkommission der Medizinischen Fakultät der LMU München, approval number 17-457, approval date September 25, 2017).

### Imaging

Both PET modalities were acquired on the same PET/CT scanning device (Biograph 64, Siemens Healthineers, Erlangen, Germany) at the Department of Nuclear Medicine of the LMU University Hospital, LMU Munich. Careful positioning including a band for fixing the head was utilized to reduce motion artifacts while avoiding patient discomfort. PET/CT scanning protocols started with a low-dose CT for attenuation correction. PET images were acquired in list mode and reconstructed using the OSEM3D algorithm with 4 iterations, 21 subsets, 5-mm Gaussian post-reconstruction filter, matrix size 168 × 168 × 109, and voxel size 2.036 × 2.036 × 2.027 mm^3^. Standard corrections were applied, which included correction for attenuation, random and scattered coincidences, dead time, and decay.

Production of [^18^F]GE-180 (*S*-*N*,*N*-diethyl-9-[2-^18^F-fluoroethyl]-5-methoxy 2,3,4,9-tetrahydro-1H-carbazole-4-carboxamide) was performed using a FASTlab synthesizer and single-use disposable cassettes (GE Healthcare, The Grove Centre, Amersham, UK) as described before [30, 34]. After intravenous bolus injection of 182 ± 15 MBq [^18^F]GE-180, the emission scan was acquired 60–80 min post-injection (p.i.) [35]. Static images were reconstructed for quantification of TSPO ligand uptake.

On another day, [^18^F]FET (O-(2-^18^F-fluoroethyl)-l-tyrosine) PET data were acquired in list mode 0–40 min after intravenous bolus injection of 179 ± 18 MBq. Dynamic [^18^F]FET PET data were reconstructed using the following time frames: 6 × 10 s, 4 × 30 s, 1 × 2 min, 3 × 5 min, 2 × 10 min. The standardized processing workflow included frame-wise motion correction to an early summation image (0–3 min p.i.) using the PMOD Fusion tool (v4.1, PMOD Technologies, Zurich, Switzerland) with standard settings for rigid matching of different modalities (human changing: trilinear interpolation, normalized mutual information dissimilarity, sample rate start 5.2 mm and final 4.0 mm, function tolerance 1.0E−4, no smoothing). This was followed by the extraction of early 5–15 min p.i. and late 20–40 min p.i. summation images.

Additionally, MRI scans were performed. This included an axial T2-weighted sequence and T1-weighted sequences before (T1_native_) and after (T1_CE_) intravenous injection of contrast agent (0.1 mmol/kg gadobenate dimeglumine, Gd-BOPTA, MultiHance; Bracco Imaging, Milan, Italy). T1_native_ images were solely used for MRI-based tumor segmentation and not for *IDH* mutation status prediction.

Before further processing and analysis of multimodal data, [^18^F]GE-180 PET and MRI images were registered and resampled to the late static [^18^F]FET PET image using the PMOD Fusion tool using the standard rigid matching settings for different modalities as specified before.

### Generation of parametric images

Static PET and MRI images were normalized as described previously using a healthy background signal [28, 36], thus yielding tumor-to-background ratio images (TBR_GE-180_, TBR_FET5-15_, TBR_FET20-40_, TBR_T1CE_, TBR_T2_). Dynamic [^18^F]FET PET information was parametrized semi-quantitatively using the time-to-peak (TTP_SUV_, TTP_TBR_) and late slope (Slope_SUV_, Slope_TBR_) of each voxel’s original and background-normalized time-activity curve (TAC_SUV_, TAC_TBR_). As described previously [37, 38], the TTP_SUV/TBR_ was estimated voxel-wise as the maximal TAC_SUV/TBR_ value after 3 min p.i. with subsequently decreasing kinetics (excluding initially high tracer concentrations in blood). According to the reconstructed time frames, the TTP categories are as follows: < 5 min, 5–10 min, 10–15 min, 15–20 min, 20–30 min, 30–40 min p.i. The late slope was derived for each voxel by linear fitting of the last three time frames (15–40 min p.i.). All resulting static and parametric images served as input for the extraction of radiomic features.

### Delineation of tumor volumes

For volume-based radiomics, the choice of segmentation method is crucial and may potentially have a huge impact on model performances. Since the chosen tumor volume highly impacts the derived radiomic features, a standardization of segmentation methods for a specific machine learning model is crucial. Therefore, we evaluated the model performances for two different tumor definition approaches: (1) tumor definition in each modality itself to be independent of the availability of other modalities and (2) whole tumor volume defined using MRI including active tumor, necrotic tissue, and peritumoral edematous/invaded tissue visible in T2/FLAIR. Tumor volumes within each modality were defined semi-automatically according to the procedure detailed in [28]. This comprises an initial manual definition of a confining volume by simultaneously taking into account information from all modalities. This volume is defined with a large safety margin around tumor tissue visible in each modality, while strictly excluding vessels, healthy ventricles, and areas outside the brain such as the skull or mucous membranes. This confining volume was applied to keep all extracted tumor volumes within the boundaries of the confining volume. The clinically established and biopsy-proven iso-contour threshold of 1.6 was applied for tumor segmentation in TBR_FET20-40_ images [39]. In analogy to TBR_FET20-40_, a threshold of 1.6 was applied for TBR_GE-180_. Since for TBR_GE-180_ no optimal biopsy-proven threshold is available, results for the additional iso-contour thresholds 1.3 and 1.8 are provided in the supplementary information. Deep learning–based delineation of tumor volumes in MRI images was performed using the BraTS toolkit [40] with the model provided by Isensee et al. [41]. Since the BraTS toolkit requires T1-weighted MRI images with and without contrast enhancement, T2-weighted MRI, and also a FLAIR sequence as input, the tool Glioma_GAN was applied for artificial generation of the missing FLAIR sequences [42]. Segmentation results comprise (a) the “active tumor” describing hyper-intensity in CE when compared to native T1-MRI and also when compared to healthy appearing white matter in CE T1-MRI; (b) necrotic (fluid-filled) and non-enhancing (solid) tumor; and (c) peritumoral edematous/invaded tissue additionally visible in T2/FLAIR [43]. The whole tumor volume, comprising areas a–c, was employed as the suspicious volume evident in FLAIR/T2 images, hereafter referred to as the “T2 volume.”

### Radiomic extraction

**Fig. 1:**
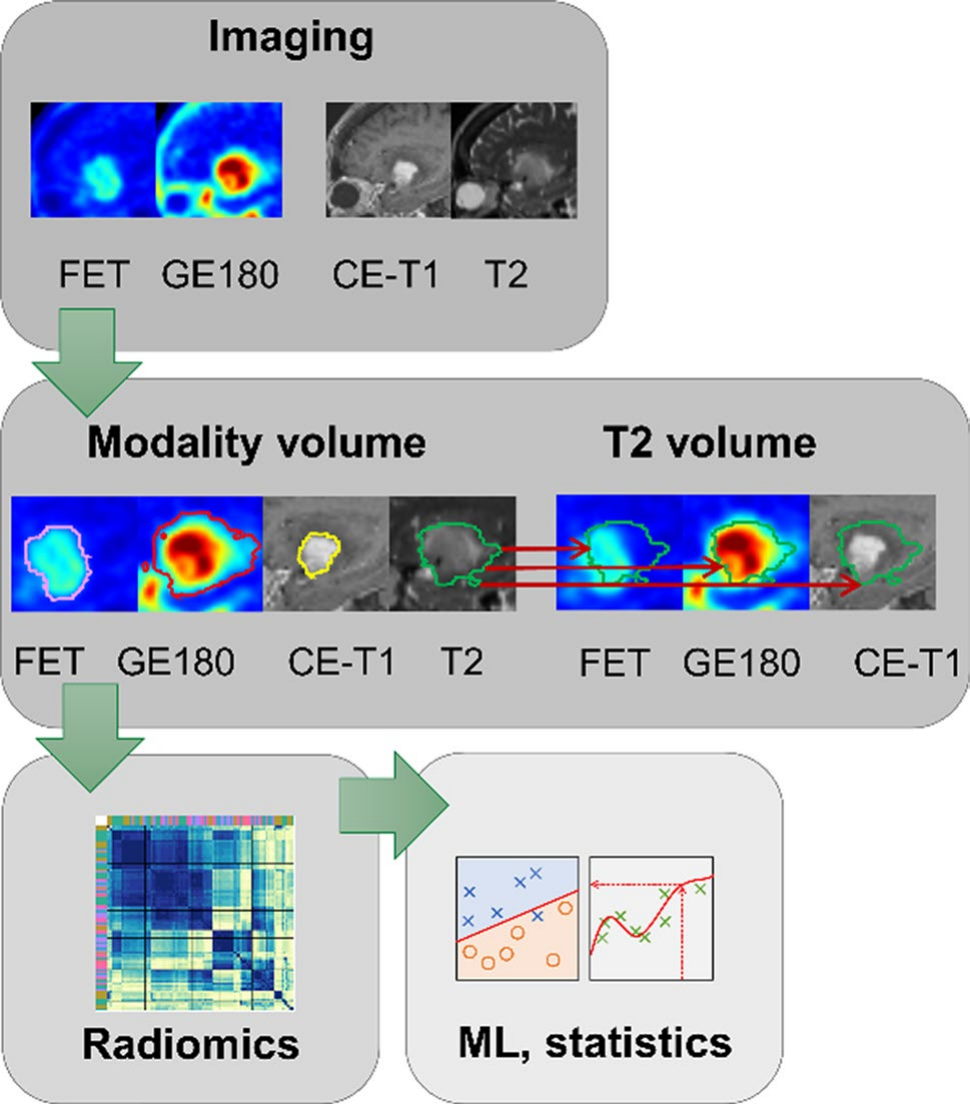
Steps of the processing workflow: (1) acquisition of multimodal data (dynamic [^18^F]FET PET, static TSPO PET, contrast-enhanced T1-weighted MRI, and T2-weighted MRI); (2) definition of tumor volumes either using suspicious volume delineated within each modality itself or applying FLAIR/T2 volumes to each modality; (3) radiomic extraction; and (4) machine learning application

Radiomics were derived from the suspicious volume delineated in each modality. This reduced the number of included patients in cases where the tumor was not visible in the respective modality. Since all gliomas tend to show an alteration in FLAIR/T2 images, while they are occasionally not detectable in PET or CE MRI, additionally, T2 volumes were applied to all modalities (Fig. 1).

Radiomic features were extracted with the Python (version 3.10) package PyRadiomics (version 3.0.1 [44]) of which most features comply with the definitions published by the Image Biomarker Standardization Initiative (IBSI) [45]. All default first-order (*n* = 18), texture (*n* = 75), and shape (*n* = 14) features provided by PyRadiomics were extracted from each modality separately. Resampling was performed to the isotropic voxel size of 2.036 × 2.036 × 2.036. The size of intensity bins was fixed for all modalities to the average interquartile range (IQR) divided by 4 [46, 47]. To take into account the typical prevalence of *IDH* wild-type and *IDH* mutant gliomas within a standard population [48], a weight of 0.6 was applied to the average IQR/4 from *IDH* wild-type gliomas, while the average IQR/4 from *IDH* mutant gliomas received a weight of 0.4. This yielded the following bin widths: 0.15 for TBR_FET5-15_, 0.13 for TBR_FET20-40_, 0.16 for TBR_GE-180_, 0.08 for TBR_T1CE_, and 0.17 for TBR_T2_. A bin width of 5.1 min was chosen for discretization of TTP values, allowing to distinguish all considered TTP categories.

### Machine learning pipeline

In addition to the evaluation of *IDH* wild-type prediction performance using the entire cohort, this study also addresses the question whether a correct classification is possible even within the subgroup of gliomas without ring enhancement on CE MRI, as ring enhancement alone is already indicative of an aggressive *IDH* wild-type glioma.

First, the individual modalities were compared using univariate and multivariate performance scores, considering radiomic features from each modality separately. Additionally, multimodal models were applied to evaluate the added value of combining data from different modalities.

**Fig. 2:**
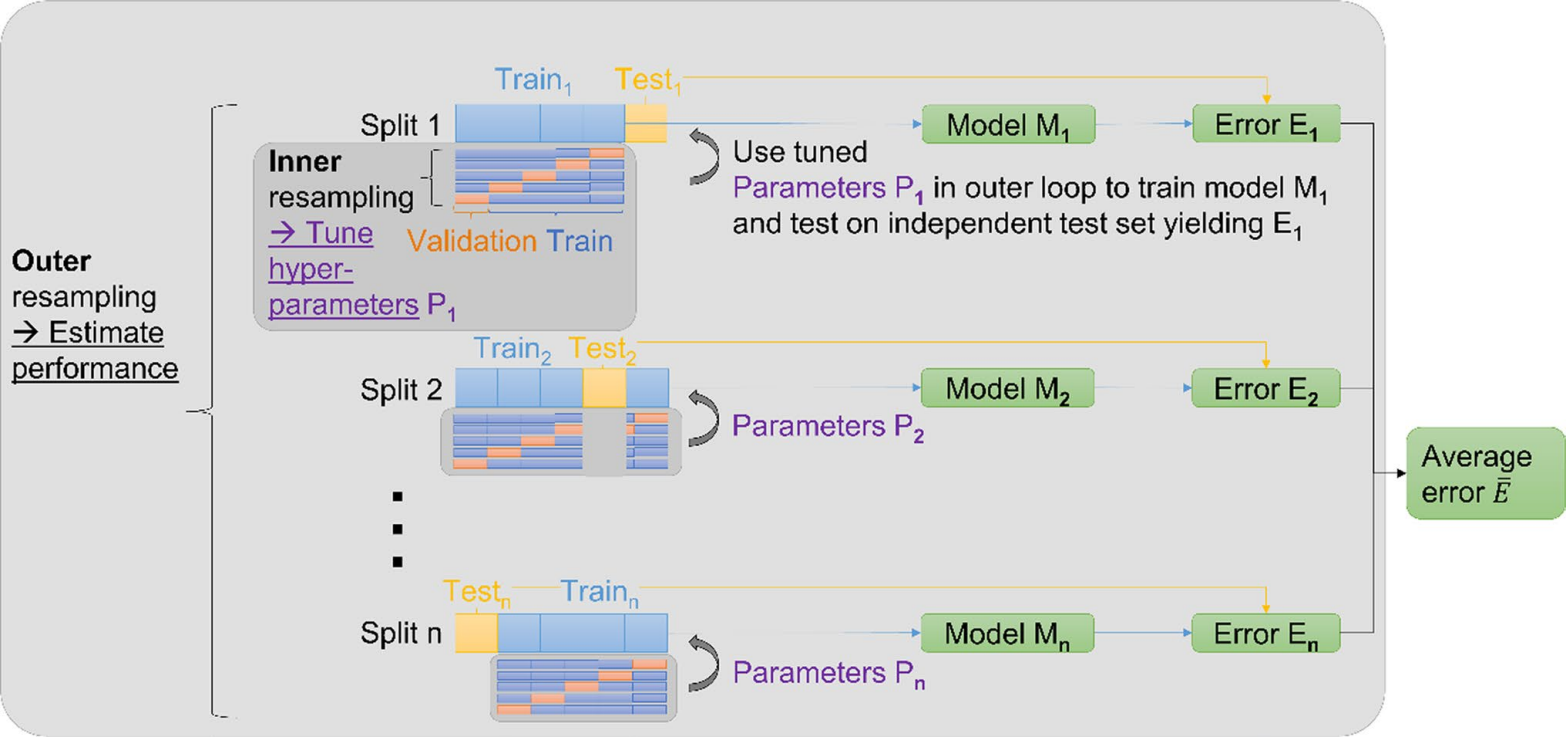
Nested cross-validation: Hyperparameter tuning is performed within the inner resampling loop using fivefold cross-validation. This yields its own optimal parameter set ${P}_{i}$ for each split $i\in \left\{1,\dots ,n\right\}$ of the outer resampling loop with $n=5\times 50=250$ for fivefold 50-repeated CV. ${P}_{i}$ is then used to train on the training set of split $i$. The trained model ${M}_{i}$ is then evaluated on the independent test set of split $i$ to yield the respective estimated error ${E}_{i}$ (i.e., performance score). The error $\overline{E }$ obtained from averaging all splits of the outer loop provides a more robust estimate of the generalization error than when only one independent test set is used

All machine learning procedures were implemented using Python (version 3.10) and scikit-learn package (version 1.2.2). Logistic regression (LR) classifiers were trained to optimize the area under the receiver operating characteristic curve (AUC). To report reliable performance measures, fivefold 50-repeated cross-validation (CV) was used. The resulting AUCs are presented as mean ± standard deviation calculated from the values of the individual CV splits. The imbalance of input data was taken into account by using stratified splits and applying LR in balanced mode. Stratification of splits results in equal distributions of the class labels in each fold, and in balanced mode, sample weights are automatically adjusted according to class frequencies. L1 regularization, employed with Liblinear solver, was utilized to prevent overfitting and to perform inherent feature selection. The LR classifiers’ other settings were left at the default values defined in scikit-learn. The machine learning pipeline comprised the following steps: (1) robust standardization of features by subtracting the median and scaling to the interquartile range; (2) exclusion of features with zero variance; (3) only for combination with clinical parameters: selection of features with nonzero LR coefficients, only for multimodal analyses: selection of *n* features with highest univariate AUC for each modality; and (4) LR classification. This pipeline was inserted into a grid search routine for hyperparameter tuning (*n* for multimodal analyses in step 3, grids 3, 5, 10; inverse regularization strength C adjusting the L1 penalty in step 4, grids 0.1, 0.3, 0.5, 0.8, 1.0) using fivefold CV within the inner loop of the nested CV scheme. Nested CV has the advantage that robust unbiased performance scores can be reported (Fig. 2). No further manual adaption of the machine learning pipeline was performed, thus ensuring no leakage of information from the test set into the comparison of modalities and multimodal models.

## Results

Patient characteristics are presented in Table 1. The subgroup without ring enhancement excluded mainly *IDH* wild-type gliomas. One *IDH* mutant WHO grade 4 astrocytoma also presented with ring enhancement in T1-weighted MRI and was therefore excluded from subgroup analyses. The AUC values of each individual model are presented in supplementary information.

**Table 1:** Patient characteristics

	Subgroup without ring enhancement	All
Number of patients	58	87
Sex (f; m)	27; 31	37; 50
Age (mean ± SD)	53 ± 16 y	57 ± 16 y
Age range	16–84 y	21–84 y
Procedure for diagnosis (biopsy; surgery)	40; 18	64; 23
*IDH* status (*IDH* wild type; *IDH* mutant)	31; 27	59; 28

### Comparison of modalities

**Fig. 3:**
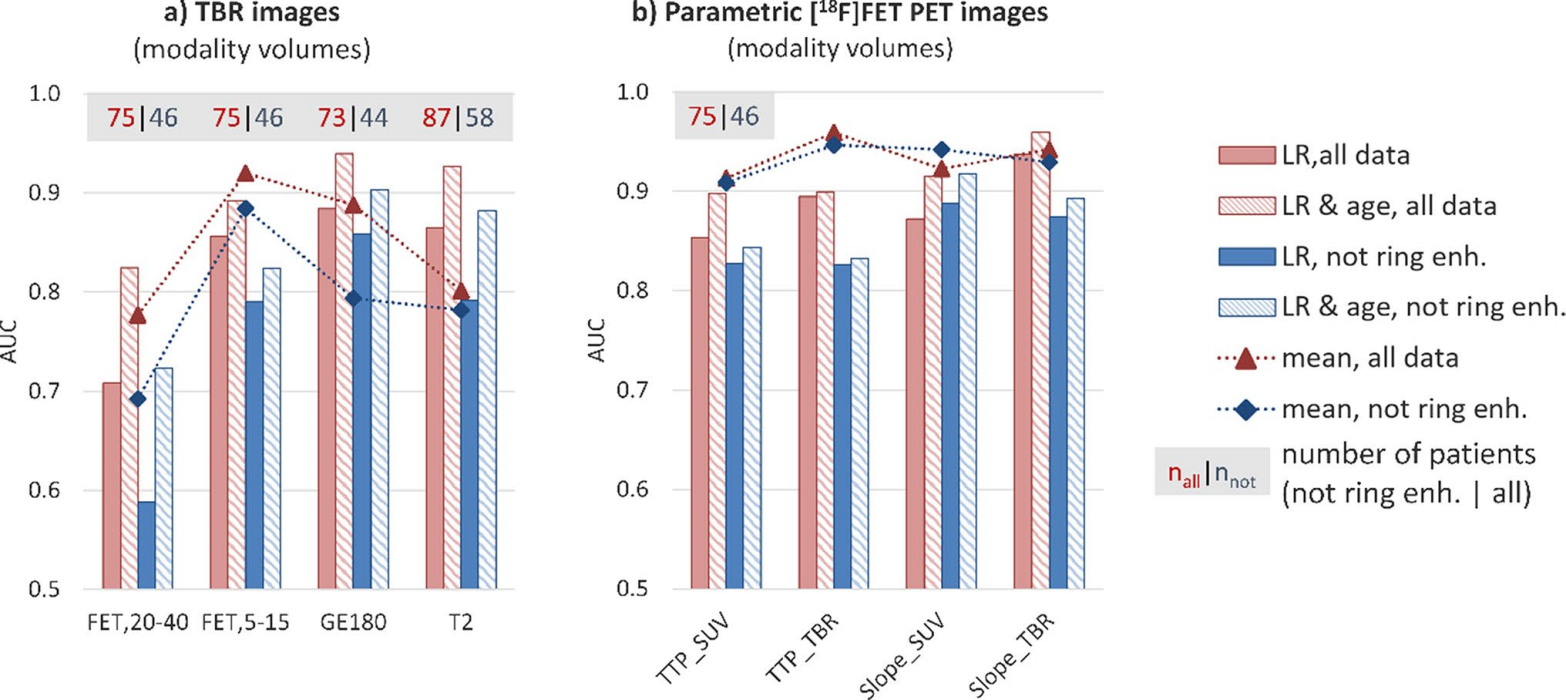
Results for TBR (**a**) and parametric (**b**) images obtained for modality volumes are presented for the entire cohort (red) and for the subgroup of gliomas without ring enhancement (blue). In addition to cross-validated AUCs from logistic regression with first-order and texture features as input parameters (plain boxes), also the logistic regression (LR) results when including age as an additional feature (dashed boxes) and the univariate AUCs obtained when using simple mean values (markers connected by dashed lines) are shown

**Fig. 4:**
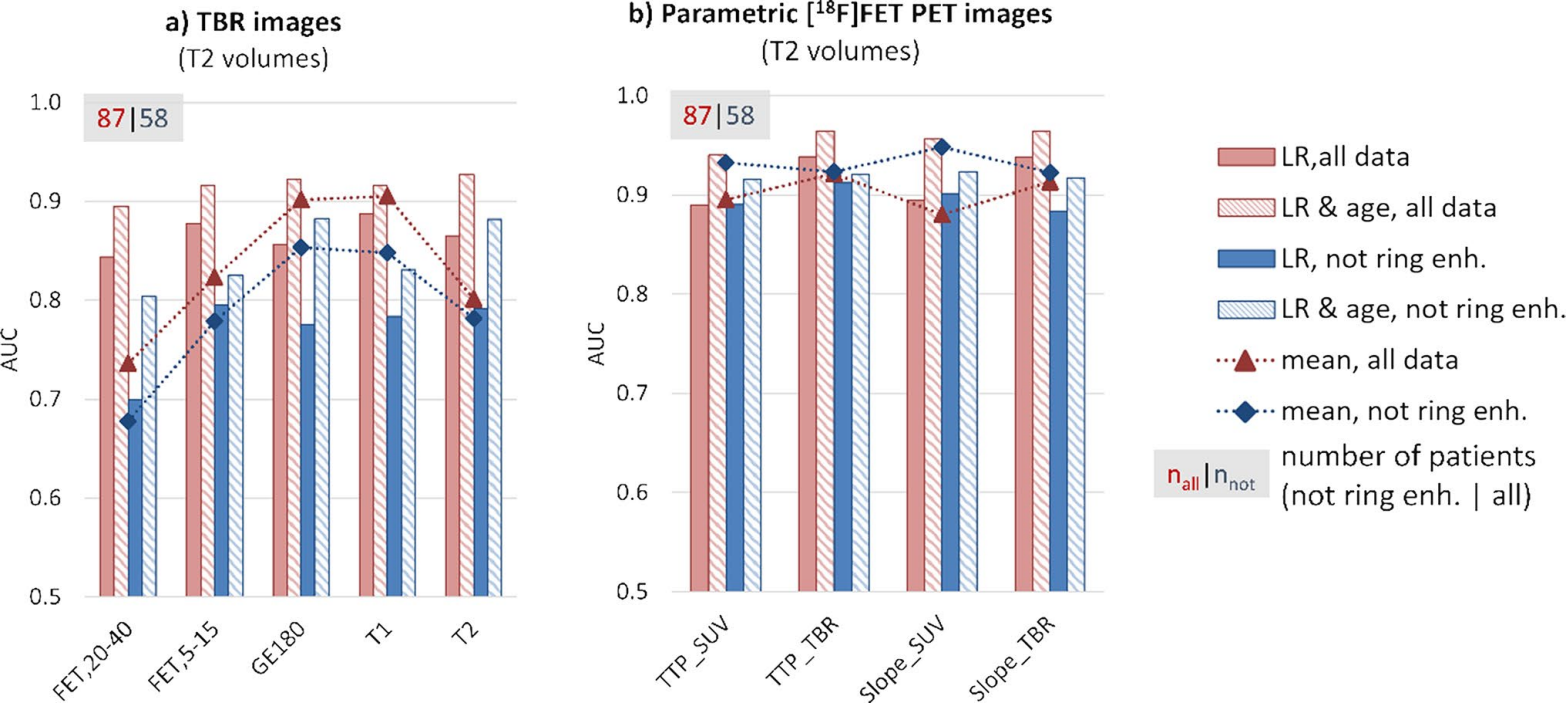
Results for TBR (**a**) and parametric (**b**) images obtained for suspicious volumes visible in T2-weighted MRI images (automatic segmentation using BraTS toolkit) are presented as described in Fig. 3

**Fig. 5:**
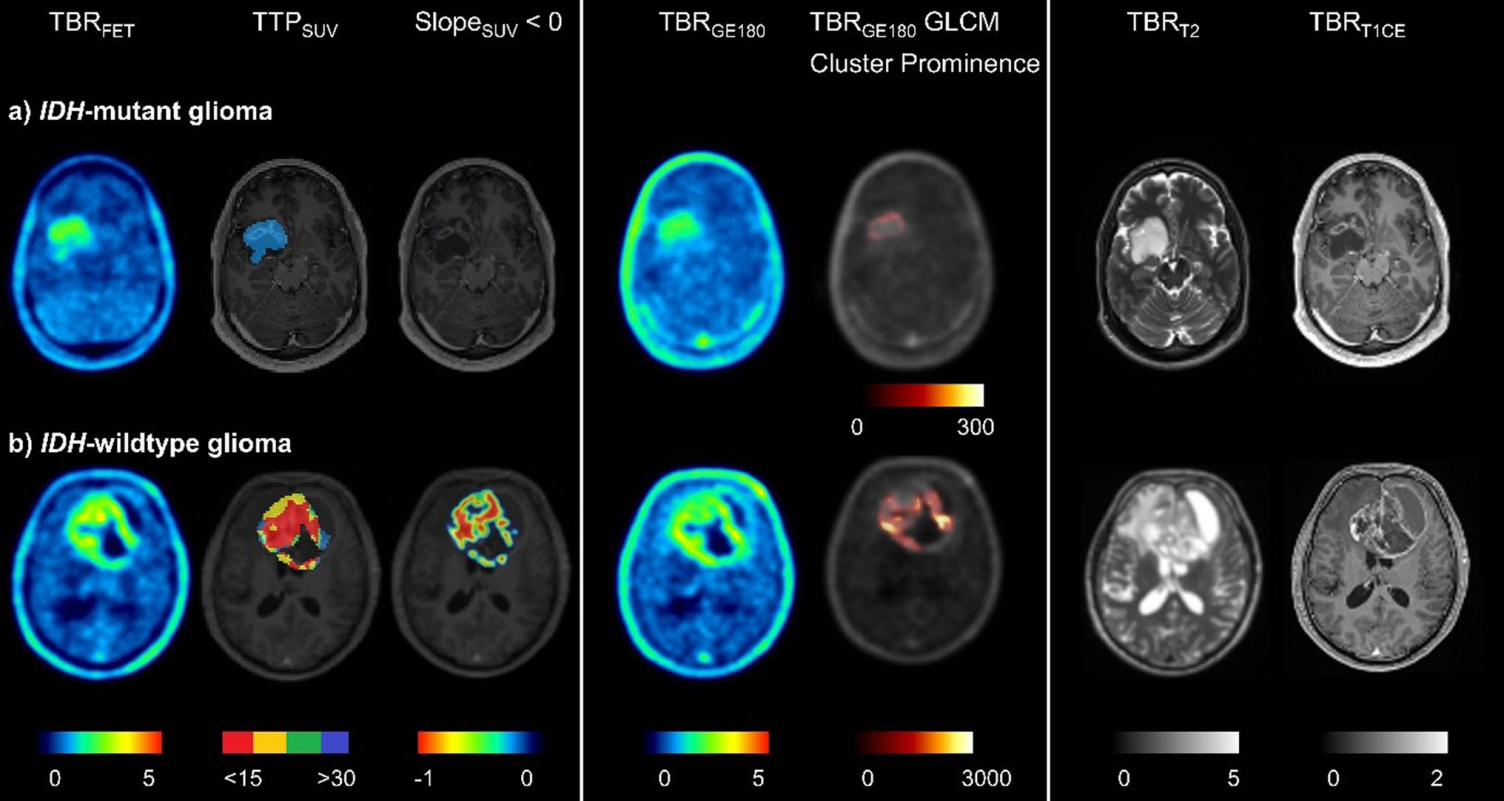
Two example patients presenting with characteristic intensity distributions within the different modalities. The *IDH* mutant glioma (**a**) shows only moderate PET uptake, an increasing kinetic (blue: TTP_SUV_ > 30 min p.i., Slope_SUV_ > 0) in dynamic [^18^F]FET PET, and a low cluster prominence as extracted from TBR_GE180_ images. The *IDH* wild-type glioma (**b**) has high PET uptake with an early peak (red: TTP_SUV_ < 15 min p.i., Slope_SUV_ < 0) and high cluster prominence. The images showing cluster prominence extracted from TBR_GE180_ images were generated using voxel-wise feature calculation of PyRadiomics with radius set to 2 voxels (kernel size 5 × 5 × 5)

A comparison of AUCs obtained for each modality is provided in Fig. 3 for modality volumes and in Fig. 4 for T2 volumes. The outcomes are displayed for the two groups: gliomas without ring enhancement and the entire cohort. Alongside the cross-validated AUCs derived from logistic regression with first-order and texture features as input parameters, we also present the LR results when including age as an additional feature, as well as the univariate AUCs obtained when only considering mean intensity as a feature. Figure 5 shows the multimodal information for one exemplary *IDH* mutant and one *IDH* wild-type glioma.

When comparing the performances of radiomic features derived from TBR images of the different modalities, LR yielded the highest cross-validated AUC for the model using TBR_GE-180_ features derived from original modality volumes (Fig. 3a) for all patients (0.88 ± 0.11) and the subgroup without ring enhancing gliomas (0.86 ± 0.12). Features derived from modality volumes of late static TBR_FET20-40_ images (Fig. 3a) resulted in the overall lowest AUC (all 0.71 ± 0.14, not ring enhancing 0.59 ± 0.20), which could be improved by the utilization of early static information from TBR_FET5-15_ images (all 0.86 ± 0.12, not ring enhancing 0.79 ± 0.16).

When further considering kinetic information (Figs. 3b and 4b), radiomic features derived from TTP and slope images reached very high LR-AUCs even when using features extracted from T2 volumes (maximal AUC for the entire cohort with TTP_TBR_ 0.94 ± 0.06, and for not ring enhancing gliomas 0.91 ± 0.08).

The performance of simple mean values (depicted by markers connected with dashed lines in Figs. 3 and 4) could be enhanced by incorporating more complex radiomic features extracted from T2 volumes in the case of TBR_FET20-40_, TBR_FET5-15_, and TBR_T2_. This improvement was also observed when evaluating the performance of features derived from TBR_GE-180_ images using original volumes. Interestingly, especially for parametric images containing kinetic information, simple mean values achieved the same high cross-validated performance as multivariate models.

Considering patient age in multivariate models of each modality significantly increased performances (dashed boxes in Figs. 3 and 4). The highest AUC was obtained for Slope_TBR_ extracted from T2 volumes for the entire cohort (0.96 ± 0.04) and for Slope_SUV_ extracted from T2 volumes for gliomas without ring enhancement (0.92 ± 0.09). The performance of the LR model using features calculated from TSPO-positive volumes of TBR_GE-180_ images reached 0.94 ± 0.08 for all patients and 0.90 ± 0.11 for the subgroup of gliomas without ring enhancement. Interestingly, the results confirmed that high age alone is already indicative of an *IDH* wild-type glioma (all 0.86 ± 0.09, not ring enhancing 0.82 ± 0.12).

Including binding affinity status did not improve the *IDH*-prediction performance of TSPO PET. Further inclusion of shape features in multivariate models could partly improve classification performance. When looking at shape features alone, features derived from TSPO PET images yielded the highest LR-AUCs (all 0.87 ± 0.08, not ring enhancing 0.79 ± 0.13).

### Sex-specific radiomic pattern analysis

Significant differences between female and male features were found especially for parameters derived from Slope_TBR_ images (Mann-Whitney *U*-test results in supplementary information). For features derived from TBR_GE180_ images, no significant differences were found for *IDH* wild-type gliomas and only few significant differences for *IDH* mutant gliomas. Additionally, the models of each modality were re-evaluated by taking into account only female or only male patients. Sex-specific analyses showed a better performance of features derived from TBR_GE180_ images using modality volumes for female patients and using T2 volumes for male patients. Average LR coefficients predominantly showed similar tendencies between female and male patients. However, for some features, the average LR coefficients differed between female and male patients (supplementary information). AUCs of the models trained on all data could not or only slightly be improved by further considering sex information as an input feature of the model. Sex alone was not able to predict *IDH* mutation status (all 0.53 ± 0.12, not ring enhancing 0.51 ± 0.13).

### Multimodal analyses

In multimodal LR analyses, the remarkable performance obtained for kinetic [^18^F]FET PET or TBR_GE-180_ parameters could not be substantially outperformed (Table 2). Multimodal analyses of parameters derived from original tumor volumes yielded an AUC of 0.97 ± 0.07 for the entire cohort and 0.94 ± 0.11 for gliomas without ring enhancement. When T2 volumes were applied, the AUCs reached 0.96 ± 0.05 for the entire cohort and 0.94 ± 0.07 for gliomas without ring enhancement.

**Table 2:** Best AUCs achieved by utilizing either first-order mean values, multivariate analyses taking into account features from a single modality without/with age inclusion, or multimodal analyses incorporating age

Patient group	All		Not ring enhancing	
Volumes from	Modality	T2 MRI	Modality	T2 MRI
First-order mean	0.96 ± 0.05	0.92 ± 0.06	0.95 ± 0.07	0.95 ± 0.06
Unimodal	0.94 ± 0.08	0.94 ± 0.06	0.89 ± 0.12	0.91 ± 0.08
Unimodal and age	0.96 ± 0.06	0.96 ± 0.04	0.92 ± 0.10	0.92 ± 0.04
Multimodal and age	0.97 ± 0.07 (*n* = 66)	0.96 ± 0.05 (*n* = 87)	0.94 ± 0.11 (*n* = 37)	0.94 ± 0.07 (*n* = 58)

The features with the highest LR coefficients differed when applying modality or T2 volumes. For modality volumes, parameters derived from TBR_GE-180_ images and the kurtosis (first order) from T2 images yielded the highest LR coefficients, followed directly by age and several parameters derived from kinetic [^18^F]FET PET images. For T2 volumes, the highest coefficients were not only dominated by parameters from kinetic [^18^F]FET PET images, but also comprised TBR_GE-180_ features, the TBR_T1CE_ feature High Gray Level Zone Emphasis, age, and features extracted from TBR_T2_ images.

## Discussion

In our study, we aimed to assess the predictive capabilities of different imaging modalities and features for identifying *IDH* mutation status in glioma patients. The results revealed that TBR_GE-180_ features extracted from TSPO-positive volumes exhibited the highest AUCs among TBR images of all modalities. This finding suggests that TBR_GE-180_ could be a reliable and promising marker for distinguishing *IDH* mutation status for TSPO-positive gliomas, i.e., 84% of all included patients. The superior performance of TSPO PET might be attributed to the fact that TSPO expression is correlated with tumor aggressiveness [49]. Moreover, aggressive glioblastomas are usually associated with an increased inflammatory component, which is depicted by the composite signal of TSPO PET primarily emanating from tumor cells within the central tumor core and increasingly from neuroinflammatory tissue within the encompassing microenvironment [27, 50]. Moreover, dynamic [^18^F]FET PET demonstrated remarkably high AUCs for parameters derived from [^18^F]FET-positive volumes (i.e., 86% of patients) and, notably, from whole tumor volumes as captured by MRI sequences (i.e., all patients). This indicates the potential of dynamic [^18^F]FET PET in providing valuable information for *IDH* mutation status prediction, not only for PET-positive gliomas but also when assessing tumors with low or even photopenic signal in PET images [51]. Our results highlight the value of static TSPO PET in predicting *IDH* mutation status, particularly in situations where *dynamic* [^18^F]FET PET data have not been acquired. While TSPO PET is not intended to replace the well-understood and well-established [^18^F]FET PET, its high performance has further been demonstrated in terms of survival prediction even among homogeneous molecular entities [25, 26]. Consequently, it emerges as a vital supplementary non-invasive tool for optimizing early patient management at the point of initial diagnosis. Similar tendencies as for the entire cohort have been found for the challenging subgroup of gliomas without ring enhancement on CE T1-weighted MRI, however, with slightly lower AUCs.

One important limitation of TSPO PET is that a superior performance is only evident when using TSPO-positive volumes. To be able to evaluate all glioma patients, also T2 volumes have been applied to each modality, where kinetic [^18^F]FET PET parameters remained highly significant. When combining features derived from TBR images using T2 volumes with age, even parameters extracted from TBR_T1_ and TBR_T2_ yielded high AUCs (all 0.92 and 0.93, not ring enhancing 0.83 and 0.88, respectively) above the level of age alone (all 0.86, not ring enhancing 0.82). This performance was slightly exceeded when features were extracted from TBR_GE-180_ images utilizing original volumes (all 0.94, not ring enhancing 0.90) and outperformed markedly when features were derived from T2 volumes applied to parametric [^18^F]FET PET images (all 0.96, not ring enhancing 0.92).

Consistent with the comparison of models for the separate modalities, multimodal analyses revealed that the highest LR coefficients comprise TBR_GE-180_ features, parameters from kinetic [^18^F]FET PET images, age, and the first-order parameter kurtosis from TBR_T2_ images. Interestingly, the inclusion of patient age in multivariate models significantly improved predictive performance. This observation highlights the potential influence of age-related molecular alterations in glioma tumorigenesis, which may further impact treatment decisions and patient outcomes.

Our results are comparable to the findings by Lohmann et al. [52], who found for a subgroup of 56 patients that standard slope (AUC = 0.74) and TTP (AUC = 0.80) parameters derived from average time-activity curves outperform simple mean values (AUC = 0.50) and texture features derived from TBR_FET20-40_ images (AUC < 0.67). With a combination of standard kinetic and texture parameters from TBR_FET20-40_ images, the maximal accuracy reached 82% using fivefold CV and cherry picking of the best parameter combination after model evaluation. Our results are also in accordance with a previously published study evaluating radiomics extracted from static [^18^F]FET PET and from several MRI sequences. Here, radiomics extracted from contrast-enhanced T1-weighted MRI images yielded a higher AUC for *IDH* prediction than radiomics from [^18^F]FET PET (0.84 vs. 0.64) and a model including all features reached an AUC of 0.79 [53]. We extended these analyses by also accounting for texture features derived from parametric images quantifying kinetic information and additionally simultaneously considering and comparing radiomics derived from TSPO PET and T1- and T2-weighted MRI images, thus reaching markedly higher *IDH* prediction performances, which also surpass the published performance of the clinically established minimal time-to-peak (0.80, no CV) [54].

Despite the promising results, our study has some limitations. The sample size may affect the generalizability of the findings, and further validation in larger cohorts is warranted. The number of glioma patients is limited due to the unique situation that simultaneous dual PET and MRI data at initial diagnosis are included. This enables a direct comparison of the different modalities using the identical patient cohort. Beyond internal validation using robust unbiased performance scores from nested cross-validation, external validation is of high importance for assessing the generalizability of the model. This applicability to other scanning devices should be addressed in follow-up studies also considering image or feature harmonization approaches [55, 56]. Moreover, larger cohorts are needed to systematically assess whether the observed differences are indeed attributable to sex-specific properties and if separate models for female and male patients are required, as suggested by Papp et al. [57]. Additionally, the choice of segmentation method for tumor delineation can influence radiomic feature extraction and subsequent predictions. Therefore, standardization and robustness of the segmentation process are essential in future studies to ensure consistency and reproducibility of results.

The ability to integrate information from multiple imaging modalities provides a comprehensive view of the tumor microenvironment, contributing to a more accurate prediction of *IDH* mutation status. This non-invasive approach not only facilitates safer and less burdensome assessments for patients but also holds promise in advancing the field of precision medicine by guiding personalized treatment strategies. Moving forward, the next step in this research would involve the application of deep learning methods, which have the advantage of leveraging entire images without the need for explicit tumor segmentation. Deep learning algorithms have shown promise in various medical imaging applications and could potentially enhance the accuracy of *IDH* mutation status prediction in glioma patients.

## Conclusions

In conclusion, our study demonstrates that TBR_GE-180_ features derived from TSPO-positive volumes, kinetic information from dynamic [^18^F]FET PET, kurtosis from T2 images, and age are important biomarkers for *IDH* prediction and even allow for an *IDH* mutation status prediction in glioma patients without ring enhancement on T1-weighted MRI. The high performance reached by non-invasive imaging holds promise for improving molecular classification and guiding personalized treatment strategies in glioma patients. Future investigations with larger and more diverse cohorts, as well as prospective validation, are necessary to establish the clinical utility of these findings.

### Supplementary Information

Below is the link to the electronic supplementary material.

Supplementary file1 (XLSX 327 KB)

## Data Availability

Detailed results can be found in supplementary material and further data are available upon request. Algorithms will be made available on GitHub https://github.com/PIANO-group.

## Abbreviations

*CE*: Contrast-enhanced
*CNS*: Central nervous system
*CV*: Cross-validation
*IDH*: Isocitrate dehydrogenase
*LR*: Logistic regression
*MRI*: Magnetic resonance imaging
*PET*: Positron emission tomography
*TAC*: Time-activity curve
*TBR*: Tumor-to-background ratio
*TSPO*: Translocator protein
*TTP*: Time-to-peak
*WHO*: World Health Organization
